# Alignment of CCI total ankle replacements in relation to midterm functional outcome and complication incidence

**DOI:** 10.1186/s13047-023-00630-2

**Published:** 2023-06-09

**Authors:** Joris P. S. Hermus, Sander M. van Kuijk, Marianne A. Witlox, Martijn Poeze, Lodewijk W. van Rhijn, Jacobus J. Arts

**Affiliations:** 1 Department of Orthopaedic Surgery, Research School CAPHRI, Maastricht University Medical Center, P. Debyelaan 25, 6229 HX Maastricht, the Netherlands; 2 Department of Trauma Surgery, Research School CAPHRI, Maastricht University Medical Center, Maastricht, the Netherlands; 3 Department of Epidemiology and Medical Technology Assessment (KEMTA), Maastricht University, Maastricht, the Netherlands

**Keywords:** Total ankle replacement, Total ankle arthroplasty, Complications, Failures

## Abstract

**Background:**

Total ankle arthroplasty is increasingly used as a treatment for end stage ankle arthropathy. The aim of this study was to report the mid-term clinical function and survival results of Ceramic Coated Implant (CCI) ankle replacements and assess the association between the alignment of the CCI total ankle replacements and early functional outcome and complication incidence.

**Methods:**

Data of 61 patients, who received 65 CCI implants between 2010 and 2016, were obtained from a prospectively documented database. Mean follow-up time was 85.2 months (range 27–99 months). Clinical function was assessed with AOFAS questionnaire and passive range of motion (ROM). Survival analysis and elaborate radiographic analysis was performed. Furthermore, complications and reoperations were recorded for all patients.

**Results:**

Progression in ROM was most seen in the first 10 months from 21.8 degrees of passive range of motion preoperative to 27.6 degrees postoperative (*p* < 0.001), while the mean AOFAS gradually increased during follow-up postoperative from a mean of 40.9 points preoperative to an average of 82.5 but shows a small decline towards the end of follow-up (*p* < 0.001). During follow-up we recorded 8 failures (12.3%) resulting in a Kaplan-Meier survival analysis of 87.7% with a median follow-up of 85.2 months.

**Conclusion:**

We observed excellent clinical results and survival after TAA with the CCI implant with only a low mid-term complication rate.

**Level of evidence:**

Level III, prospective cohort study.

## Introduction

End-stage arthritis of the ankle joint can substantially disrupt the Quality of Life (QoL) of a patient through functional limitations [1, 2]. Total ankle arthroplasty (TAA) and ankle arthrodesis (AA) are the two primary surgical treatment options for patients who fail conservative measures. Development of the total ankle arthroplasty is challenging because of the small contact area and high forces which can lead to high contact stresses [3–5].

Ever since TAA surgeries have been undertaken for osteoarthritis, patient satisfaction, pain relief and function have changed for the better [6], however, potential risks of only small improvement of range of motion, persistent pain, and low functional outcome following TAA still remain high [7–9]. TAA may be more susceptible to complications, failure and subsequent re-operation as compared to other surgical interventions of the ankle, especially compared to ankle arthrodesis [10]. For example, a study conducted by Spirt et al. [11] observed that 28% of the patients that underwent ankle arthroplasty were re-operated due to complications with a follow-up time of 5 years. Watts et al. [12] documented in their systematic review a 2.5 times higher re-operation rate in TAA compared to ankle arthrodesis. Nonetheless, the perioperative major complications in ankle arthrodesis occurred 1.8 times more often but had a 29% lower risk of a minor complication after adjusting for patient and hospital factors, such as gender, age, and health-status [13]. These findings are confirmed by the higher 30-day re-admission rate for ankle arthrodesis, which has an independent risk factor with an odds ratio of 2.51 [14].

Salvage arthrodesis after failed ankle arthroplasty has an overall complication rate of 18.2% whereas the overall non-union rate was 10.6%, and it has also inferior results to primary arthrodesis [15, 16]. Therefore, minimal bone resection is necessary in the surgical procedure of TAA to enhance the union rate [17].

Until January 2023 there were no non-designer studies presenting short-term clinical results of exclusively the CCI ankle prosthesis. The group of Doets et al. reported two designer studies. First report presented both the Buechel-Pappas and the CCI ankle prosthesis showing a survival of 87% in the post fracture group and 79% in the instability group at 6 years of follow-up [18]. The other designer study reported a survival rate of 67.5% at 10-year follow-up, with a complication rate of 54% and 37% patients underwent reoperation [19].

This is the first non-designer study to report the mid-term clinical results of the Ceramic Coated Implant (CCI) ankle replacements. The aim of this study was to report the first clinical results and survival of 65 consecutive CCI ankle replacements with a median follow-up time of 85.2 months. Secondly, to report the complication and failure rate.

Thirdly, to ascertain if associations could be observed between implant migration during follow-up, as measured according to the Rippstein protocol [20], and subsequent failure of the implant.

## Material and methods

Sixty-five consecutive CCI total ankle replacements were placed between June 2010 and June 2016 and included in this prospective follow-up study. In January 2021, the last clinical follow-up was performed. The study was approved by the local ethical committee of Maastricht University (METC 2018-0709), and all patients provided written informed consent prior to participation in the study. All TAA’s surgeries were performed by a non-designer single surgeon (JH) in Maastricht University Medical Centre (MUMC +), using the CCI ankle replacement (Wright Medical Technology, Arlington, TN, USA). The importance of single surgeon studies may allow better comparison, because of consistent diagnosis and different experience levels of surgeons.

The following standard surgical protocol and standardized post-op rehabilitation was followed. Postoperative patients had an aftertreatment with a cast for 2-3 weeks, 50%weightbearing till 6 weeks and from 10 weeks onward, the patients were allowed to advance to full weightbearing. Achilles’ tendon lengthening was performed when perioperative an ankle dorsalflexion of 10 degrees couldn’t be reached. Additionally, these patients received a cast as aftertreatment for 6 weeks. Afterwards these patients followed standard aftertreatment protocol. Thromboembolic prophylaxis with low-molecular-weight heparin was given until 6 weeks postoperative. Patients were assessed pre-op and postoperatively at 1 day, 6 weeks, 3-6-12 months and thereafter yearly. The American Orthopedic Foot and Ankle Score (AOFAS) questionnaire and passive range of motion (ROM) were assessed. During every follow-up moment all complications and re-operations were recorded, Radiographic evaluation was performed at follow-up moments of 6 weeks, 3-6-12 months and thereafter yearly according to the Rippstein protocol [20]. Failure of the implant was defined as the need of revision surgery of one of the metal components or (pan)arthrodesis, according Henricson et al. [21]. They define the term revision as removal or exchange of one or more of the prosthetic components except for incidental exchange of the polyethylene insert (e.g., due to infection).

**Fig. 1:**
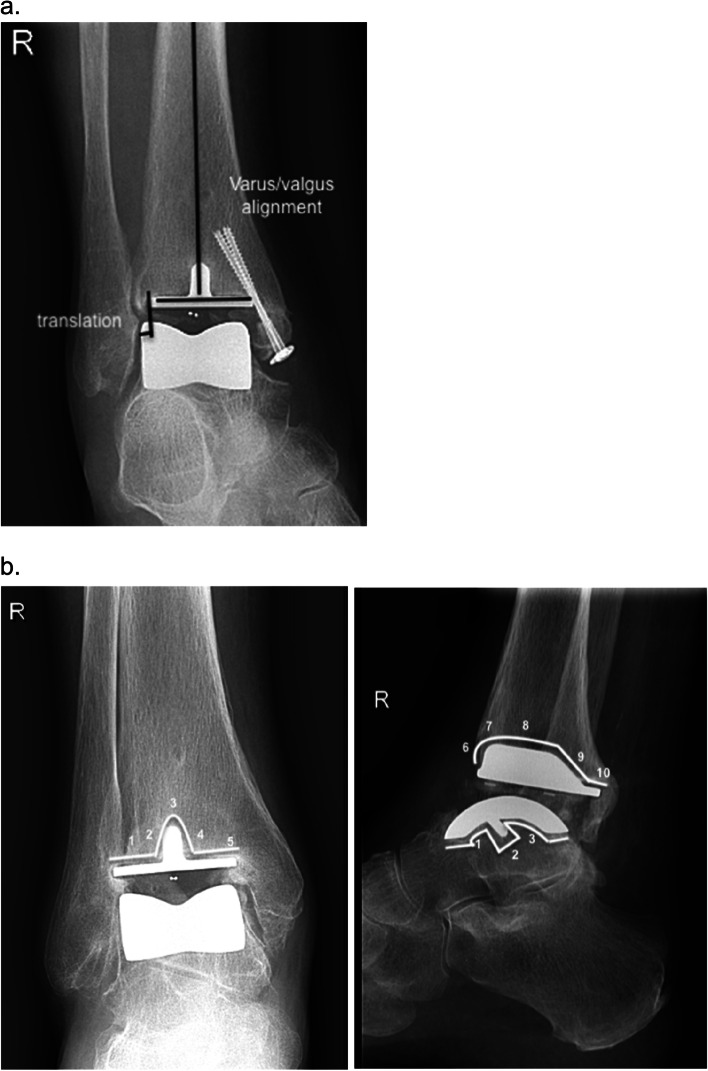
Visualisation of X-ray angle measurement for alignment and migration assessment according to Rippstein protocol (18). **a** Measurement varus/valgus aligment and translation. **b** Zones of radiolucency

Radiographic Evaluation assessment consisted of the estimation of prosthesis alignment, migration, translation, and radiolucent lines using the Rippstein protocol (Fig. 1) [20]. These assessments were evaluated by two independent reviewers (JV, PV) who did not perform an operation on any of the patients.

Standing anteroposterior (AP) and lateral (LAT) radiographs of the ankle were made preoperatively and at each follow-up visit. Ankle alignment was determined on the anteroposterior radiograph by measuring the angle between the talar and the tibial component. An angle of 0° is considered ideal, and > 5° was defined as varus or valgus ankle alignment compared to the tibial anatomical axis. The posterior slope of the tibial component was measured on the lateral radiograph. The components were considered to be improperly aligned on the anteroposterior radiograph if the medial or the lateral border of the talar component extended more than 2 mm beyond the border of the tibial component.

Tibial radiolucencies > 1 mm in width were assessed in five zones around the implant on the anteroposterior radiograph and in five zones on the lateral radiograph. Talar radiolucencies were assessed in three zones around the implant on the lateral radiograph. Migration of the tibial component was defined 121 as a change of an angle of > 3°. Migration of the talar component was defined as > 2 mm of subsidence into the talar bone. In addition, we recorded the presence and location of periprosthetic cysts and osteophytes [20].

### CCI design rationale

**Fig. 2:**
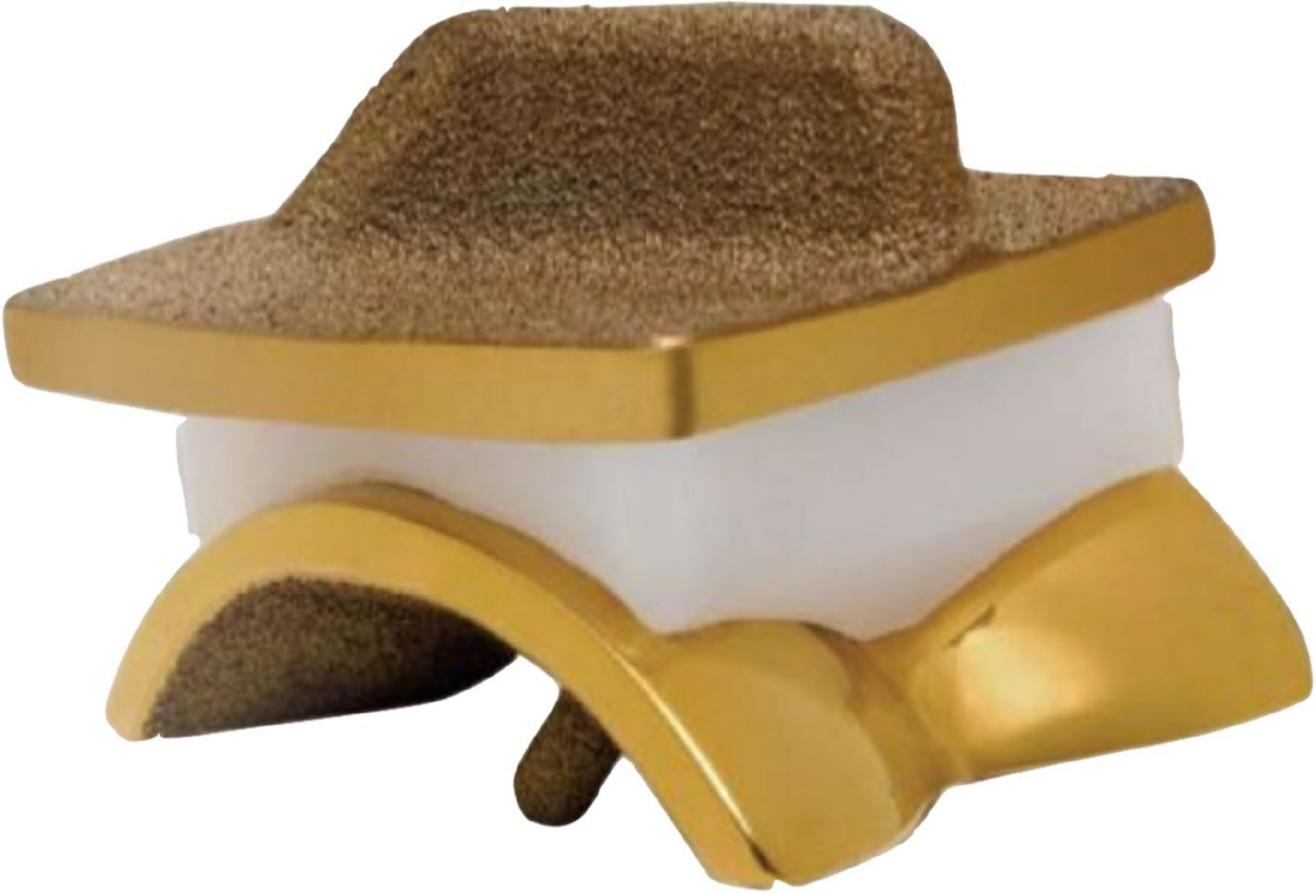
The CCI Evolution mobile-bearing prosthesis design

The CCI Evolution mobile-bearing prosthesis design (Wright Medical Technology, Arlington, TN, USA) combines three principles: Preservation of bone stock, cement free fixation and reduction of abrasion (Fig. 2). The three components, including mobile bearings, allow minimal bone resection of the tibia as well as of the talus. The tibial component requires only a 2.8 mm resection of the distal tibia. The talar component, with its triple V-design, will requires a resection as little as possible.

The CCI Evolution prosthesis has a trapezoid tibial component with the same AP ratio of 1.4 to 1 as the human tibial surface. In contrast to the Buechel-Pappas prosthesis, the CCI ankle uses a fin instead of stem on the tibial side. The talar component requires a triple-V-shaped resection of the talar dome instead of a curved resection.

The cement-free fixation is made possible by a titanium surface (CoCrMo metal) with a plasma spray coating of a biomimetic BONIT®calcium phosphate coating with a thickness of 20 ± 10 μm and a Ca/P ratio: 1.1 ± 0.1 (DOT, Rostock, Germany). The metal parts of the CCI Evolution ankle prosthesis are coated with a titanium nitride (TiN) coating. The design of the talar component includes a deep sulcus, conforming the distal articulating surface of the polyethylene bearing, providing medial-lateral stability.

### Statistical methods

Patient baseline characteristics were described as mean and standard deviation (SD) or median and range for continuous variables that were distributed normally or were skewed respectively. Categorical characteristics were summarized as count and percentage. Similar descriptive statistics were used to describe characteristics of the procedures, complications, and clinical outcomes. To quantify a potential learning effect, the ankles were dichotomized into the first half by calendar time and the second half. Subsequently, Fisher’s Exact test was used to test for a difference in occurrence of perioperative complications.

A Kaplan-Meier curve was used to assess the cumulative survival of the ankle replacement. As a sensitivity analysis, a second Kaplan Meier curve was plotted including also insert changes as failures.

## Results

### Patient and demographics

Between April 2010 and June 2016, a single foot and ankle surgeon (JH) performed 65 consecutive primary TAA’s with the CCI implant in 61 patients. Gender distribution was 18 female (29.2%) and 43 male (70.8%), with a mean age at first ankle replacement of 65.4 years (± 7.3, range: 52.5 to 81.7). The median follow-up time was 85.2 months (range 54.1 – 128.4 months). One patient had a failure of the total ankle replacement within seven months of follow-up because of migration of the tibia component caused by a postoperative fracture. The demographic information of the study cohort is displayed in Table 1. Adjacent joint disease was present in 53 171 (81.5%) of the 65 ankle joints.

**Table 1:** Patient baseline characteristics

Characteristic	Total cohort (*N* = 61)
Gender	
female	18 (29.2%)
male	43 (70.8%)
Age at first procedure (years)	65.4 (7.3)
BMI (kg/m^2^)	27.4 (3.6)
Bilateral ankle replacement	4 (6.6%)
Side^a^	
right	38 (58.5%)
left	27 (41.5%)
Indication for operation^a^	
arthritis	51 (78.5%)
rheumatoid arthritis	8 (12.3%)
hemochromatosis	4 (6.2%)
hemophilia	1 (1.5%)
tuberculosis	1 (1.5%)
Follow-up time (months)	47.8 (24.5)
Adjacent disease^b^	49 (80.3%)
talonavicular joint	13 (21.3%)
subtalar joint	27 (44.3%)
Chopart	16 (26.2%)
midfoot	6 (9.8%)
other	3 (4.9%)
Subtalar arthrodesis prior to procedure	2 (3.3%)
Double arthrodesis prior to procedure	3 (4.9%)

Data are presented as mean (sd) or count (percentage)

^a^Summary over all 65 procedures

^b^Sum of categories is larger than the total number of affected patients due to multiple locations in some patients

Six weeks prior to the TAA some cases required additional surgery, such as subtalar (3.1%) and double arthrodesis (7.7%). The time frame was chosen to reduce the immobilization time in a plaster.

On pre-operative conventional standing X-rays, a mean varisation was seen of 10.0 (SD = 3.4) degrees in 23 (35.4%) ankles on the anteroposterior view, and valgisation of 8.9 (SD = 2.6) degrees in 11 (16.9%) ankles. A perioperative medial malleolus osteotomy was necessary in 9 out of 65 procedures (13.8%) because of ligament balancing caused by a varisation of the talus pre-operative according to the technique described by Doets et al. [22]. Additional Achilles tendon lengthening was performed in 28 of 65 ankles.

### Clinical results and functional outcomes

**Fig. 3:**
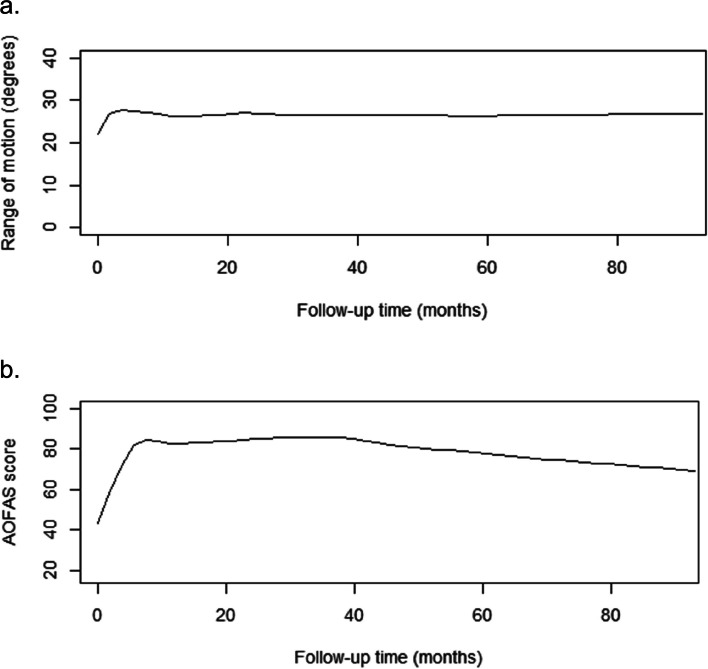
**a** Range of motion during follow-up. **b** AOFAS during follow-up

After TAA, the Range of Motion improved preoperative from mean 21.8 degrees (SD = 9.0) to 27.6 degrees (SD = 7.0) at final follow-up postoperative (*p* < 0.001). The AOFAS hindfoot score improved significantly from a mean of 40.9 (SD = 12.9) points preoperative to an average of 82.5 (SD = 13.1) over the whole follow-up period of 7.1 years (*p* < 0.001). Figure 3 shows the postoperative changes of both the Range of Motion and the AOFAS score. The most progression in ROM was observed in the first 10 months while the AOFAS gradually increased till 16 months postoperative.

### Per-operative complications and failures

Ten intra-operative complications (15.4%) of which 7 were medial malleolar fractures (70.0%) were observed. One or more post-operative complications occurred in 23 ankles (35.4%). Impingement (8 ankles 196 (12.3%)) and deep infection (7 ankles (10.8%)) were the most frequently recorded. All the perioperative complications occurred in the first thirty surgeries, none occurred in the later surgeries. Twenty-three ankles (35.4%) required one or more re-operations. Gutter impingement was the primary reason for a reoperation in 4 ankles (4 revisions to a thicker UHMWPE insert of the 7 impingements (57,1%)).

During follow-up, we recorded 8 (12.3%) failures and 6 insert changes (2 because of a DAIR procedure in case of an infection [23] and 4 for impingement). Of those 8 failures, 6 were (pan)arthrodesis, and 2 ankles required revision surgery of all components. The median time to failure was 12.2 months of follow-up (IQR 6.1, 14.9).

### Survival analysis

**Fig. 4:**
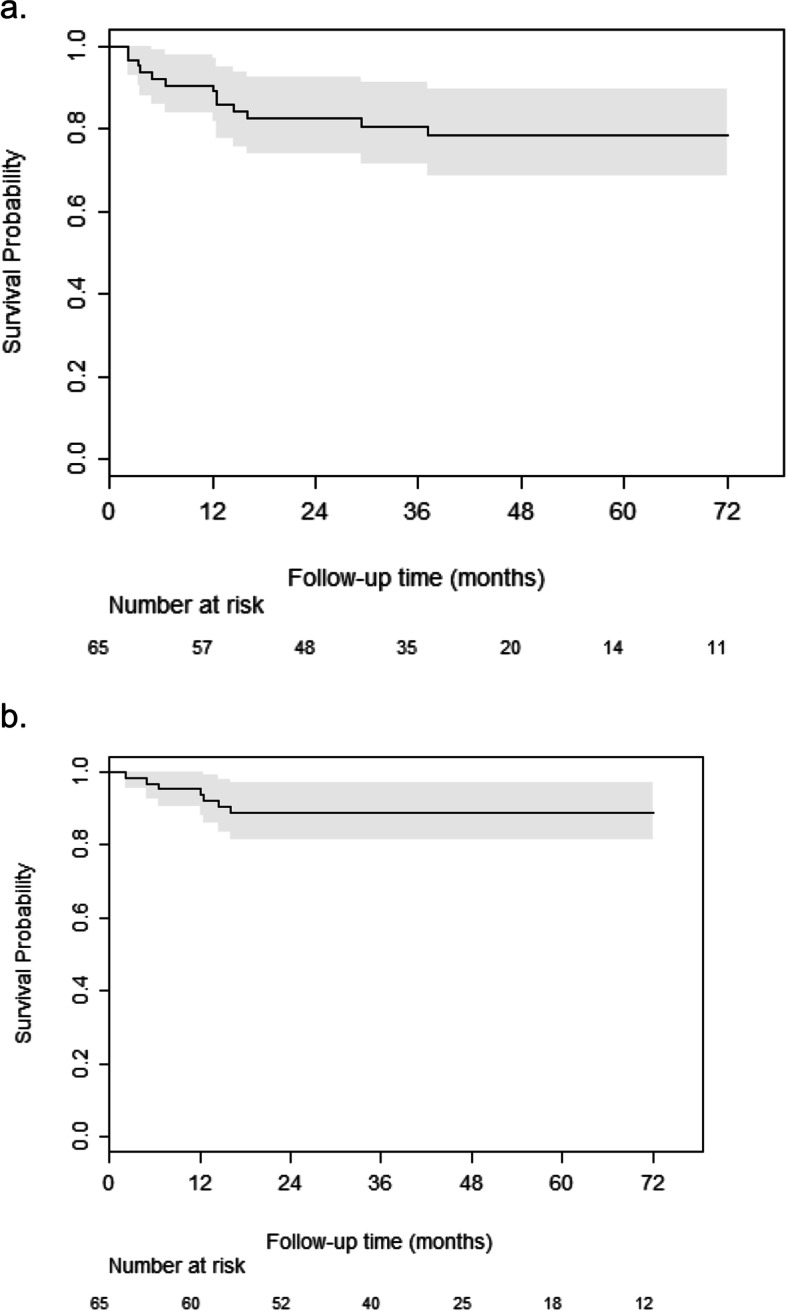
**a** Kaplan -Meier curve of the survival probability of an ankle implant, including insert change as failure. **b** Kaplan-Meier curve of the survival probability of an ankle implant, disregarding insert change as failure

Kaplan–Meier curves of the survival probability of ankle replacements excluding and including insert changes are shown in Fig. 4. Kaplan-Meier survival analysis of the CCI ankle implant at mid-term follow-up showed a survival rate of 87.7% with a median follow-up of 85.2 months. Table 2 shows associations between patient characteristics before surgery and failure of the TAA. Due to the small number of failures, we were unable to estimate the association between valgisation/varisation and the risk of failure. We did observe a significant association with pain duration and the risk of failure (HR = 0.87, 95% CI: 0.78 – 0.97).

**Table 2:** Associations between characteristics before surgery and subsequent failure after surgery

Characteristic	Hazard ratio (95% CI)	*p*-value
Varisation (degrees)	1.19 (0.28-5.10)	0.811
Valgisation (degrees)	u.e	u.e
AOFAS hindfoot score	1.02 (0.97 – 1.08)	0.449
Weight (kg)	1.02 (0.96 – 1.09)	0.512
BMI (kg/m^2^)	1.14 (0.96 – 1.34)	0.136
Diabetes Mellitus	0.88 (0.11 – 7.33)	0.908
Pain duration (years)	0.87 (0.78 – 0.97)	**0.013**
ASA score	0.86 (0.26 – 2.81)	0.803

*u.e* unable to estimate, *CI* confidence interval, *BMI* body mass index

### Radiologic outcomes

Radiologically, 11.3% of the TAA’s were positioned in varus (mean 6.5 degrees; SD = 1.2) and 0% in valgus. Migration in the frontal plain was measured on conventional X-rays in three ankles and in the sagittal plane in two ankles respectively.

Radiolucency is significantly increasing with the follow-up time (*p* = 0.02). At the mean follow-up of 85.2 months 54 patients had radiolucency of more than 1 mm, of which only 15 in the talus. No trends were seen as to how much radiolucency occurred in the talar or tibial component.

According to the Rippstein protocol radiolucency was not seen in 11 of the 65 ankle replacements (16.9%). The radiolucency zone 3 (Fig. 1), for the CCI ankle replacement, was associated higher risk for failure; HR 6.4 (95% BI:1.4, 29.0; *p* = 0.0152). On the last follow-up with a mean follow-up of 85.2 months the X-ray showed in 15 ankles (23.1%) a cyst in the talus with a mean diameter of 17.8 cm (range 8.9-29.5 cm) and in 13 ankles 20%) in the tibia with mean diameter of 15.2 cm (range 9.1-22.1 cm). In total, cyst formation in ankle replacement was found in 26 of 65 ankle replacements (40%). Only in two failures cyst formation was found in the talus. No significant correlation was found between failure and varus /valgus malpositioning or a gap between the bone and prosthesis.

## Discussion

In this study, we report the first mid-term clinical outcome results of the Ceramic Coated Implant (CCI) ankle replacements after a median follow-up time of 85.2 months. The functional outcome "Range of Motion", improved after ankle replacement from 21.8 preoperative to 27.6 degrees postoperative. The AOFAS hindfoot score improved significantly from a mean of 40.9 points preoperative to an average of 82.5 over the entire follow up period of 7.1 years. Valderrabano et al. [24] showed that these changes in gait were accompanied by a significant improvement in AOFAS, SF-36 and ROM [24]. The results of the CCI Evolution Total Ankle System, presented in this study, can be compared to the designer study of Doets [17]. In this study, the 5-year follow-up results of 75 CCI ankle prosthesis and 15 Buechel-Pappas ankle prostheses showed a survival rate of 87% in the postfracture group and 79% in the instability group [18]. Recently the group of Doets reported even a lower survival rate of 67.5% at 10-year follow-up [19].

In this non-designer study the reported Kaplan-Meier survival analysis of the CCI ankle implant at mid-term follow-up showed a survival rate of 87.7% with a median follow-up of 85.2 months. 1,226 prostheses were analyzed in the Swedish Ankle Registry with a mean follow-up of 7 years. An overall survival rate at 5 years was found of 0.85 (95% CI 0.83-0.87), at 10 years 0.74 (CI 0.70-0.77) [25] which is comparable with our presented results.

TAA is an emerging treatment and might be an alternative to ankle arthrodesis in the treatment of end-stage ankle arthroplasty. However, there are also disadvantages to this intervention. TAA may be more sensitive to complications, failure and subsequent re-operations compared to ankle arthrodesis [26]. Simonson et al. [27] showed that 44,2% of 2453 total ankle replacements had a complication. These complications could lead to failure. Usuelli et al. [28] showed that most of the operative variables as well as clinical and radiological outcomes stabilized after a surgeon had performed 28 cases. We have reported all our perioperative complications in our first thirty ankle replacements. The median time to failure was 12.2 months of follow-up (IQR 6.1, 14.9). Kamrad et al. [29] showed that mean time from primary TAR to revision surgery was about two years.

Intra-operative complications occurred in 10 ankles (15.4%) and one or more post-operative complications in 23 ankles (35.4%). Excluding the failures, there were 15 postoperative complications in the remaining 57 ankles (26.3%). The most postoperative complications were impingement and deep infection. In the 8 impingements a gradual postoperative coronal translation of the talus was seen 7 cases with a mean translation of 3.8 mm. In the group of impingements, the median was (IQR): 3.0 (2.2-5.0) compared to the group without impingement 271 (IQR): 2.2 (0.0 – 3.4). Similar complication and reoperation rates were found in the designer study which presented results of the Buechel-Pappas and CCI prosthesis [18]. The 10-year follow-up study of the same group 45% of the total ankle replacements had developed a complication of which only 16% was treated conservatively [19]. Causes for impingement could be component malrotation that will always lead to gutter impingement [30, 31]. Nunley et al. [32] noticed that the reoperation rate was higher in mobile-bearing total ankle replacements compared to fixed bearing total ankle replacements, and in most cases to relieve impingement.

In this study, it was also assessed if associations could be observed between implant migration during follow-up, as measured according to the Rippstein protocol [20], and subsequent failure of the implant. According to the Rippstein protocol analysis, progressive radiolucency was seen in 8 ankle replacements (12.5%). The radiolucency zone 3, for the CCI ankle replacement, was associated with a higher risk for failure; HR 6.4 (95% BI:1.4, 29.0; *p* = 0.0152). Therefore, we advocate to specify these values for TAA patients on X-ray evaluation during clinical follow-up.

The association between total ankle component alignment and biomechanical contact stresses and clinical outcome has been extensively studied [6, 8–11, 33–37]. Pyevich et al. [38] found higher rates of pain in tibial components placed in more than 4° of valgus and 19 cases of migration of a component of TAA. We could not find a significant correlation between failure and varus /valgus malpositioning or a gap between the bone and prosthesis.

In the prospective cohort study of Dalat et al. [39] 29.8% had symptomatic cysts which required treatment at a mean 59.8 months. Of which 16.7% of their patients were treated by curettage and grafting, 13.9% ended up in an arthrodesis. Najefi et al. [40] reports that in 78% of the patient’s bone cysts were not removed by implant resection, of which 30% of the cases were larger than 5 mm. The description of osteolysis as the first “mechanical” type of radiolucency is probably incorrect, as this most likely refers to the presence of pre-existing cysts that had not been observed, or progression of pre-existing cysts due to stress shielding. We reported that 40% of our 65 ankle replacements in our cohort had cyst formation while only two failures showed cyst formation.

This study has a number of limitations that have to be considered. First, this study includes a relatively small number of patients. Making generalizations from a small sample size is a cognitive bias. Second, the AOFAS score combines a clinician-reported and patient-reported parts. Forty of the possible 100 points are determined by clinical examination and, therefore, the evaluator can be biased when grading the clinical results [41]. Third, the learning curve of the CCI Evolution Total Ankle System was part of this prospective follow-up, as we have reported all our perioperative complications in our first thirty ankle replacements. Finally, this study reports on the CCI TAR, a prosthesis that was withdrawn from the market in 2016 by Wright Medical Group (Memphis, USA). However, mid-term outcomes of this 3-component prosthesis remains relevant and will contribute to the limited literature available.

## Conclusion

The mid-term results of the CCI Evolution Total Ankle System cohort are comparable to literature of other TAA designs with a survival of 87.7% at 7.1 years of follow-up [18, 25].

Functional recovery is two-fold with most progression in ROM seen in the first 10 months, while the mean AOFAS gradually increased during follow-up postoperative.

Additional studies with larger cohorts and longer follow-up are warranted to determine which patient specific factors, surgical technique and designs are a higher risk for failure to improve the patient satisfaction, clinical outcome and survival rate of ankle replacement.

## Data Availability

The datasets used during the study are available from the corresponding author on a reasonable request.

## Abbreviations

CCI: Ceramic Coated Implant
AOFAS: American Orthopaedic Foot and Ankle Society
ROM: Range of Motion
TAA: Total Ankle Arthroplasty
AA: Ankle Arthrodesis
METC: Medical Ethics Assessment Committee
AP: Anteroposterior
LAT: Lateral
TiN: Titanium nitride
SD: Standard deviation
SF-36: Short Form Survey-36
